# Novel Aspects of Toxicity Mechanisms of Dioxins and Related Compounds

**DOI:** 10.3390/ijms21072342

**Published:** 2020-03-28

**Authors:** Raimo Pohjanvirta, Matti Viluksela

**Affiliations:** 1 Department of Food Hygiene and Environmental Health, University of Helsinki, Mustialankatu 1, FI-00790 Helsinki, Finland; 2 School of Pharmacy (Toxicology) and Department of Environmental and Biological Sciences, University of Eastern Finland, P.O. Box 1627, FI-70211 Kuopio, Finland; matti.viluksela@uef.fi; 3 Environmental Health Unit, Finnish Institute for Health and Welfare (THL), P.O. Box 95, FI-70701 Kuopio, Finland

**Keywords:** 2,3,7,8-Tetrachlorodibenzo-*p*-dioxin, TCDD, dioxins, halogenated aromatic hydrocarbons, polycyclic aromatic hydrocarbons, aryl hydrocarbon receptor, toxicity mechanisms, developmental toxicity

## Abstract

Dioxins and related compounds are common environmental contaminants. Although their levels have gone down, they are still of concern, in particular regarding developmental toxicity. The adverse effects of these compounds are mediated by the aryl hydrocarbon receptor (AHR), whose canonical signaling pathway has been unveiled in fair detail. The alternative (non-genomic) pathways are much more obscure. AHR has also proven to be a master regulator of numerous physiological phenomena, which has led to the search of selective AHR modulators with low toxicity. Papers of this Special Issue address the developmental toxicity of dioxins and related compounds as well as selective modulators of AHR and both its canonical and alternative signaling pathways. In addition, new optical and stereoscopic methods for the detection of dioxins are presented. As a whole, this Special Issue provides an up-to-date view on a wide variety of aspects related to dioxin toxicity mechanisms from both original research articles and reviews.

Dioxins have been fascinating toxicologists for over 50 years because of their unique toxicity spectrum and a specific binding protein, the aryl hydrocarbon receptor (AHR), which mediates their pathophysiological effects but whose primary endogenous ligand is still obscure. The AHR was discovered in mid-70s as the mediator of induction of xenobiotic-metabolizing enzymes by dioxins and polycyclic aromatic hydrocarbons (PAHs) such as 3-methylcholanthrene (3MC) [1]. It further proved to be indispensable for the toxicity of the dioxin prototype molecule, 2,3,7,8-tetrachlorodibenzo-*p*-dioxin (TCDD) [2], and important, if not essential, to that of PAHs [3]. The AHR was found to be a ligand-activated transcription factor, whose canonical signal transduction mechanism (as exemplified by induction of CYP1A1) has since been elucidated in fair detail. Much less is known about alternative (non-genomic) signaling routes and their biological significance.

Activation of the canonical pathway results in altered expression of numerous genes regulated by the AHR. One of these genes is *Tiparp*, which encodes a mono-ADP-ribosyltransferase. This enzyme acts as an AHR repressor [4]. Previous studies by Matthews et al. have revealed that global knockout of TiPARP in mice renders them highly susceptible to TCDD-induced steatohepatitis, wasting syndrome, and lethality [5], and that TiPARP knockout in mouse hepatocytes alone suffices to increase sensitivity to these toxicities of TCDD [6]. In the present Special Issue, this research team reports on the effects of a non-dioxin-like AHR agonist, 3MC, in globally TiPARP-deficient mice [7]. Although the knockout animals were more sensitive to the acute lethality of the test compound, the cause of death differed from TCDD; TiPARP-deficient mice exposed to 3MC accumulated substantial volumes of viscous fluid in their abdominal cavities. The liquid met all but one (neutrophils vs. lymphocytes) of the criteria for chylous ascites in humans. Thus, although TiPARP deficiency enhances the toxicity of both TCDD and 3MC, their modes of action appear to be distinct, if not divergent.

Overall, individuals are at their most sensitive to dioxin toxicity during their early development. Several studies published in this Special Issue focus on this stage. Using human embryonic stem cells, Sarma et al. [8] studied the developmental effects of TCDD on the neural differentiation system, with special reference to dopamine-producing neurons. They found a temporal window of sensitivity at the embryoid body stage, with exposure to TCDD on day 9 leading to increased sprouting of neuronal progenitors and expression of tyrosine hydroxylase (rate-limiting enzyme of dopamine synthesis) at both mRNA and protein levels, whereas exposure on either day 0 or 35 had no effect. Another study employed zebrafish to delineate the transcriptomic responses to PAHs. Zebrafish have three AHR isoforms: AHR1a, AHR1b, and AHR2. Of these, AHR2 and AHR1b bind TCDD while AHR1a mediates the toxicity of some PAHs. Shankar et al. [9] assigned PAHs to eight classes based on their developmental toxicity profiles in zebrafish and assessed representatives of these classes for their transcriptomic effects prior to the manifestation of overt toxicity. They then constructed a network linking the PAHs based on coordinated gene responses elicited by them. The PAHs analyzed constituted two clusters, of which the one deviating more from controls consisted of PAHs with generally more severe developmental toxicity and higher *Cyp1a* expression. Especially in the skin, Cyp1a protein expression seemed to be mediated by AHR2. While all PAHs of this cluster predominantly activated AHR2, they also each enriched unique pathways, suggesting that molecular events downstream of AHR2 ramified among the PAHs.

The developmental toxicity of TCDD to laboratory animals, predominantly zebrafish and mice, is reviewed in the Special Issue by Yoshioka and Tohyama [10]. They provide evidence that both cardiac and skull malformations induced by TCDD in zebrafish are mediated by AHR2 and critically involve SOX9b. They also show that TCDD-triggered hydronephrosis in mice is actually not a single entity but two separate syndromes. The fetal form of it arises as a corollary of ureter obstruction, whereas the neonatal form (caused by TCDD exposure during the first couple of days after birth) is due to neonatal stage-specific upregulation of prostaglandin E2 synthesis and mediated by polyuria. Ventral lobe agenesis is a characteristic feature in TCDD-induced abnormal prostate development. The authors state that the suggested mechanism is altered WNT/β-catenin signaling cascade caused by AHR activation in the urogenital sinus mesenchyme, which disturbs the budding of the sinus epithelium. The review further elaborates on TCDD-induced cleft palate, concluding that although it is temporally and morphologically well characterized, its molecular basis is still elusive.

Despite the fact that the production of polychlorinated biphenyls (PCBs) has been banned for some 40 years by now, these compounds continue to be ubiquitous in the environment due to their persistence, release from PCB-containing equipment, and inadvertent arise of PCBs as byproducts of contemporary pigment and dye production. Developmental neurotoxicity is a primary endpoint of concern associated with PCB exposures. Klocke and Lein [11] review behavioral and mechanistic data obtained from experimental models as well as recent epidemiological studies on PCB developmental neurotoxicity. They posit that of the 209 PCB congeners, it is not the 12 dioxin-like coplanar compounds (usually considered the most toxic of PCBs) that are mainly responsible for developmental neurotoxicity, but the non-dioxin-like congeners, including the lower chlorinated PCBs (≤4 Cl atoms). Importantly, the PCB doses used in many of the animal studies described in the review were found to result in tissue levels that are within the range observed in contemporary humans.

The possibility that dioxins could cause adverse effects in unexposed future generations was originally raised by findings from Michael Skinner’s lab [12,13,14] and has subsequently gained support by studies demonstrating the ability of dioxins to cause epigenetic alterations. Two reviews address these points. Viluksela and Pohjanvirta [15] recount key studies assessing the multi- and transgenerational impacts of dioxins. They pay special attention to the lowered male/female ratio in the offspring after paternal dioxin exposure, because there is epidemiological evidence to suggest that this effect may also occur in humans and at exposure levels not too far off the prevailing background concentrations. Patrizi and Siciliani de Cumis [16], in turn, give an overview of the main mechanisms by which environmental factors may interfere with the epigenome (DNA methylation, histone modifications, non-coding RNAs), followed by description of the reported effects of TCDD on each of them. They emphasize the importance of future studies to examine the stability of pollutant-induced changes in the epigenome in the course of a lifetime and how epigenetic changes in germ cells may be transmitted across generations.

Two original research reports probe further the role of non-genomic pathways in the toxicities of AHR ligands. In the canonical pathway, AHR dimerized with AHR nuclear transporter (ARNT) binds to the DNA at dioxin response elements (DREs) with a defined core consensus sequence (5’-T/NGCGTG-3’). However, Vogel et al. had previously revealed that AHR can physically interact with the NF-κB subunit RelB and bind to an unrecognized RelB/AHR responsive element of the IL-8 promoter [17]. Here, they used RelB knockout mice and various AHR agonists to characterize in more detail the role of RelB in AHR-regulated immune responses [18]. They made the highly interesting finding that both AHR agonist-induced and basal expression of the cytokines IL-17A and IL-22 and the chemokine CCL20 required the functional expression of RelB. Furthermore, the tryptophan-derived AHR agonist FICZ increased more IL-17A and IL-22 mRNA expression than TCDD. The outcome of co-exposure with lipopolysaccharide depended on the AHR agonist. In another study, Lagadic-Gossmann et al. [19] examined the effects of extractable organic matter from diesel-exhaust particles on intracellular calcium concentration and membrane microstructure in endothelial cells. They found that the lipophilic n-hexane (containing most of the PAH compounds extracted) and dichloromethane extracts rapidly increased intracellular calcium concentration via AHR non-genomic signaling (as verified with an AHR antagonist and siRNA), while the more polar methanol and water extracts had no effect. Moreover, the dichloromethane extract appeared to induce an AHR-dependent reduction in the global plasma membrane order.

As AHR is a major physiological regulator of, e.g., the immune system, a lot of effort is currently put into attempts to discover selective AHR modulators with low toxicity. In this Special Issue, Prokopec et al. [20] report on the transcriptomic effects in rat liver of a promising novel AHR agonist (IMA-08401) that resembles laquinimod, an immunoregulatory drug in phase III clinical trials. The immunomodulatory effect of laquinimod is attributable to AHR activation, which is likely due to a minor metabolite of it, deethylated laquinimod. The prodrug IMA-08401 is converted to this metabolite to a far greater extent than laquinimod. Earlier studies had shown that although IMA-08401 is a strikingly potent inducer of AHR-regulated xenobiotic-metabolizing enzymes in vitro, it has low toxicity in vivo in rats, probably because of its short elimination half-life [21,22]. Here, it was found that acute exposure to IMA-08401 leads to changes in transcription of genes involved in antiviral and antibacterial responses, while subacute exposure causes an oxidative stress response in rat liver. In both cases, the transcriptomic profile differed remarkably from that of TCDD.

AHR plays a pivotal role in skin barrier integrity by upregulating the expression of epidermal barrier proteins including filaggrin (FLG), loricrin (LOR), and involucrin (IVL). In addition to AHR, another important regulator of keratinocyte proliferation and differentiation is the OVO-like 1 (OVOL1) transcription factor. Since certain phytochemicals are known to modulate AHR and/or OVOL1 activities, Hashimoto-Hachiya et al. [23] studied here the extract of *Rhodiola crenulata*, which contains several AHR agonists. The extract induced *CYP1A1* gene expression in human keratinocytes. It also upregulated FLG, LOR, IVL, and OVOL1 mRNA and protein expression levels in an AHR-dependent manner. However, whereas FRG and LOR proved to be influenced by both AHR and OVOL1 activities, IVL was only regulated by AHR. These finding are relevant to atopic dermatitis in which FLG, LOR, and OVL are all downregulated in the lesions and restored by appropriate treatments. This extract has thus potential as a novel remedy for that skin disease.

Finally, Patrizi et al. [24] review new techniques for detection and real time monitoring of dioxins and related compounds in gaseous or liquid phases based on optical and spectroscopic methods. The gold standard method for analytical evaluation has long been gas chromatography combined with high-resolution mass spectrometry. Of the new methods, plasmonic sensors enable non-invasive analysis and offer astonishingly high sensitivity (down to a single molecule), but at present this may occur at the expense of selectivity. Mid-infrared devices are under development and may allow in vivo measurements of dioxins and related compounds in the not-too-distant future.

Overall, these 12 papers of the Special Issue cover a wide range of aspects broadly related to the toxicity of dioxins and the underlying mechanisms. While they present recent achievements of research in this field, they concomitantly bring up gaps that still exist in our understanding of these phenomena. Bridging the gaps in the future will be important not only toxicologically, but also physiologically because of the multitude of essential roles the AHR plays in the organism. Although emissions and environmental levels of dioxins have decreased substantially during the last four decades, for the most sensitive adverse effects the intake of dioxins is still close to or even exceeds their tolerable weekly intake level in all age groups in Europe [25]. This fact gives additional significance and urgency to studies on dioxin toxicity mechanisms.

## Abbreviations

AHR	Aryl hydrocarbon receptor
PAH	Polycyclic aromatic hydrocarbon
3MC	3-Methylcholanthrene
TCDD	2,3,7,8-Tetrachlorodibenzo-*p*-dioxin
PCB	Polychlorinated biphenyl
ARNT	AHR nuclear transporter
DRE	Dioxin response element
FLG	Filaggrin
LOR	Loricrin
IVL	Involucrin
OVOL1	OVO-like 1
